# Brief internet-based cognitive behavior therapy program with a supplement drink improved anxiety and somatic symptoms in Japanese workers

**DOI:** 10.1186/s13030-017-0111-y

**Published:** 2017-09-01

**Authors:** Kentaro Shirotsuki, Yuji Nonaka, Jiro Takano, Keiichi Abe, So-ichiro Adachi, Shohei Adachi, Mutsuhiro Nakao

**Affiliations:** 1 Faculty of Human Sciences, Faculty of Human Relations, Musashino University, 3-3-3, Ariake, Koto-ku, Tokyo, 135-8181 Japan; 2 Suntory Global Innovation Center Research Institute, Kyoto, Japan; 3 Medical Corporation So-bun-kai, Clinic Adachi, Gifu, Japan; 4 Department of Psychosomatic Medicine, Teikyo University Hospital, Tokyo, Japan

**Keywords:** Cognitive behavior therapy, Internet, L-carnosine, Self-help, Workplace

## Abstract

**Background:**

Self-help cognitive behavior therapy (CBT) is a useful approach for the treatment of psychological problems. Recent research on the effectiveness of self-help internet-based CBT (ICBT) indicates that the paradigm moderately improves psychological problems. Furthermore, previous studies have shown that food and drinks containing supplements improve various health conditions. We investigated the effect of a brief self-help ICBT administered with a supplement drink on psychological well-being and somatic symptoms.

**Methods:**

In total, 101 healthy workers were enrolled in the 4-week ICBT program, which consisted of psychoeducation on stress management, behavior activation, and cognitive restructuring. The supplement soft drink was taken every day during the program. The participants were instructed to watch on-demand video clips and read the self-help guidebook and supporting comic strip weekly on the Internet or smartphone. The Japanese version of the Profile of Mood States (POMS) was administered before and after completion of the program. Scores on the POMS tension-anxiety (POMS-TA), depression (POMS-D), and fatigue (POMS-F) subscales were used to assess the effect of the program. Somatic symptoms were assessed using the Brief Job Stress Questionnaire.

**Results:**

In total, 75 participants continued the program for 4 weeks; however, of those, 27 failed to complete all weekly tasks or meet the post-assessment deadlines. Therefore, the data of 48 participants were included in the analysis. Pre-post intervention comparisons using paired *t*-tests revealed significant improvement on the POMS-TA, but not the POMS-D or POMS-F subscales. Moreover, participants reported a significant reduction in the severity of low back pain.

**Conclusion:**

Our brief intervention moderately improved anxiety levels and the symptom of low back pain. These findings suggest that the brief ICBT program is effective in non-patient populations. Future directions for brief ICBT are discussed.

**Trial registration:**

This study was registered on February 10, 2016 at UMIN. The registration number is UMIN000020962.

## Background

Recently, evidence-based behavioral healthcare has been provided over the Internet, via computers, tablets, or smartphones. Traditionally, self-help treatment has involved the use of printed manuals or books [1, 2]; however, several Internet and computer-based self-help treatment programs have been developed and tested recently. Internet-based cognitive behavior therapy (ICBT) is a technology-based CBT technique delivered with or without the support of a clinician. The findings of previous meta-analyses indicate that computer-based psychological treatments for depression [3] and anxiety disorders [4] are effective. Furthermore, meta-analyses suggest that self-help CBT is effective for the treatment of low-to-moderately severe psychological problems [5, 6]. Self-help treatments, such as ICBT, can overcome barriers to care such as limited availability of clinicians trained in evidence-based interventions [7]. Moreover, ICBT is effective for patients who are reluctant to seek medical help because of stigma [8]. However, a previous study found that technology-based treatments had a high dropout rate [9].

Previous research suggests that work-related psychological problems are associated with depression and anxiety disorders [10, 11]. The psychophysiological function of workers is affected by increased work pace, more high-skilled jobs, and an increase in the use of information and communication technology [12]. Previous studies suggest that distinct demanding or threatening extrinsic features of job environments and employment conditions may be detrimental to workers’ health and that specific personality traits and coping behaviors may increase workers’ vulnerability to stress [13, 14]. Furthermore, chronic job-related stress has been shown to adversely affect physical and mental health and increase the risk of cardiovascular diseases and impair work performance [15]. Suwazono et al. [16] found that the threshold for working hours associated with fatigue symptoms under the worst job-related stress was very close to the standard daily working hours in Japan. The negative effects of long working hours on subjective fatigue have been documented in Japanese workers [17, 18], highlighting the importance of preventing the development of stress-related psychological problems by reducing work-stress factors.

Several recent studies have examined the effects of web-based CBT programs for employees. Kimura et al. [19] found that a brief CBT-based training program significantly improved subjective work performance. An investigation of the combined effect of brief CBT education with web-based CBT homework in healthy workers found that the program moderately alleviated symptoms in employees with clinically significant psychological distress [20]. These reports suggest that self-help CBT is a moderately effective intervention for a range of common mental health problems in the work place.

Recent research has investigated the effect of consuming food with supplements on health conditions. Some studies have focused on carnosine, which is present in meats such as chicken and beef. Carnosine is immediately absorbed intact in the jejunum, despite being a dipeptide [21]. It is metabolized by the enzyme carnosinase [22] and excreted via the kidneys [21]. Carnosine is decomposed into β-alanine and histidine in the blood, and it is considered to be effective in recovery from fatigue. Carnosine has L-carnosine and D-carnosine. In the present study, we used L-carnosine because it is a natural ingredient, but D-carnosine is not. L-Carnosine is a naturally occurring amino acid found in high concentrations in muscle, heart, and brain tissues. We felt that by conducting research with supplements close to natural ingredients, it would be possible to examine the situation in a manner close to everyday life.

A previous study found that consumption of a drink containing imidazole dipeptides (400 mg) over an 8-week period significantly improved fatigue compared with a placebo [23]. Yamano et al. [24] found that daily intake of a chicken extract that contained large amounts of imidazole dipeptides (carnosine and anserine) promoted recovery from mental fatigue. Anserine (beta-alanyl-1-methyl-L-histidine) is a methylated form of carnosine that is present at high levels in the breast skeletal muscle of chicken. Imidazole dipeptides are natural antioxidants in meat extract. The antioxidant effect of imidazole peptide inhibits tissue damage and suppresses the reduction in performance level induced by mental fatigue. These findings suggest that foods or beverages containing imidazole dipeptides may be used as anti-fatigue supplements [24]. A previous study that examined the effect of computerized CBT, which included both Internet based and off-line software based programs, in combination with a supplement drink containing L-carnosine found that the program improved anxiety and fatigue [25]. In contrast, little is known about the effect on workers of brief self-help ICBT combined with a supplement drink.

Thus, based on previous findings, this study was done to test our hypothesis that the combination of easy-to-implement self-help CBT and supplementation with L-carnosine would improve mental health, in terms of anxiety, depression, and fatigue, in the workplace.

## Methods

### Participants

The participants were healthy volunteers who were full-time employees at beverage, alcoholic beverage, and food manufacturing/sales companies in Kyoto, Japan. Participants included the employees of the company that created the supplement drink. Members directly belonging to the division related to this research did not participate in this study. The present study was a preliminary study that comprised a single arm of an investigation into the effect of ICBT administered with a drink containing supplements. Due to the nature of the intervention, participants were aware of their allocation status.

The exclusion criteria included systolic blood pressure less than 90 mmHg; pregnancy, possible pregnancy or lactation; current participation in other studies; the presence of internal diseases; history of cardiovascular disease or diabetes mellitus; individuals with a soy allergy, because soy related material was included in the supplement drink; and investigator-determined unsuitability. The Japanese version of the Profile of Mood States (POMS) scale [26] was administered to all participants. Participants with scores above the criteria scores of the clinical group on the POMS were excluded from research participation in advance due to the risk of mental disease. This included males with a POMS tension-anxiety score of 28 or more and females with a score of 30 or more and males with a POMS depression score of 34 points or more and females with a depression score of 37 points or more. These scores represent the cut-off for clinical classification.

Participants were recruited through poster and Internet web page advertisements. An advertisement was posted in the office, and those who wanted to participate were able to access the research participation HP. An Internet web advertisement was sent by e-mail to employees who expressed a wish to participate. Potential applicants accessed the research HP listed in the e-mail. Initially, 113 individuals responded to the advertisements; however, seven who did not agree with the purpose of the study declined to participate. Thus, 106 participants were enrolled after providing informed consent on the website before the start of the program. An additional five participants were disqualified because they failed to watch the preparatory psychoeducation video, leaving 101 participants registered in the program. Those who were excluded were urged to continue treatment. When a high POMS score was found, the person was automatically excluded from participation, but information on our in-house health service or a supervisor who could be consulted was provided.

**Fig. 1:**
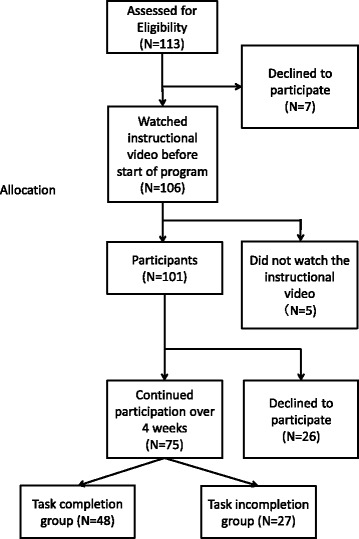
Participant flow chart. The flow chart shows the progression of participants throughout the study. First, 113 people accessed the registration web page to participate. Before the start of the program, an informed consent session was held in a web forum where the study purpose and procedure were explained. One-hundred and six participants provided web-based informed consent to participate in the study. However, five participants were disqualified because they failed to watch the preparatory psychoeducation video. In total, 75 participants completed the 4-week program, and of those, 48 completed all of the weekly tasks. The data of the 48 completers were included in the analysis. We conducted a supplementary analysis to examine differences between participants who did and did not complete all of the tasks

Of the 75 participants (74%) who completed the 4-week program, 27 (27%) failed to complete the weekly tasks or meet the post-assessment deadline (less than 80% completion of the tasks). Therefore, the data of 48 participants (47%, 35 males and 13 females; mean age, 36.02 years ±9.73 SD) were included in the analysis of the per protocol set. The participant flow chart is shown in Fig. 1.

### Protocol and ICBT program

The participants watched a video clip on mental health and completed questionnaires before and after the 4-week program. Participants received four weekly installments of an ICBT program consisting of psychoeducation about stress management, stress coping, behavior activation, and cognitive restructuring on their computer or smartphone. The program was completely devoid of therapist contact or support and was delivered using an on demand e-learning system. A supplemental web textbook, based on a self-help guidebook titled “Stress ni makenai hon (in Japanese)” [27], was provided.

Participants watched weekly e-learning video clips (5–10 min) and read the corresponding sections of the guidebook during the 4-week intervention. A supporting comic strip that contained supplemental content from the textbook was provided daily, and participants were able to read the content as needed. After watching the video clips and reading the textbook, participants were instructed to perform weekly tasks on the website. Additionally, participants were instructed to record their mood on a scale of 1 to 5 daily. The content of the ICBT program is shown in Table 1. An original website was created for the present ICBT program. Participants accessed this site and carried out all the practices and records included in this program, such as questions, daily records, weekly tasks, and videos clips. This website could be accessed anywhere from terminals that can use the Internet, such as mobile phones, smart phones, and PCs.

**Table 1:** Internet-based cognitive behavioral therapy program content

Week 1	Psycho-education about stress models
Week 2	Stress-coping
Week 3	Behavioral activation
Week 4	Creating your own cognitive model

In case of an adverse event, the protocol was to consult the investigator and take appropriate measures, as necessary. Follow-up surveys were to be carried out until the individual recovered or improved; however, no adverse events occurred during or after the study.

### Supplement drink

Participants were instructed to consume one bottle (190 mL) of a soft drink that mainly contains L-carnosine (200 mg). We hypothesized that L-carnosine may have an effect on the improvement of anxiety, depression, and fatigue. In addition, this drink included Fructose glucose liquid sugar, citric acid (anhydrous), trisodium citrate, DL-malic acid, (ST) sucralose, acesulfame K, soy related material, flavors, and carbon dioxide. Participants took this drink every morning during the 4-week intervention and were asked to record consumption of the drink on the website daily. The contents of the soft drink were not disclosed to the participants before the start of the program. This drink was not a commercial item, but it was created for this research.

### Measures

#### The Profile of Mood States (POMS)

The Japanese version of the POMS was administered before and after completion of the program, and the tension-anxiety (TA), depression (D), and fatigue (F) subscales were assessed using T-scores (standardized scores).

The Japanese version of the POMS has high reliability and validity, with reliability coefficients (Cronbach’s alpha) of 0.779–0.926 for the factors [26]. Scores ≥34 and 20 are the cutoffs for depression and mild depression, respectively, for males, and scores of ≥28 and 18 are the cutoffs for anxiety and mild anxiety, respectively [28]. Furthermore, the POMS is a reliable and valid measure of mood states in older adults [28].

#### The Brief Job Stress Questionnaire English version [29]

The Brief Job Stress Questionnaire (BJSQ) consists of 57 items measuring work stressors, somatic symptoms, and social support for workers on a 4-point Likert-type scale. The BJSQ has high internal consistency (Cronbach’s alpha) and construct validity [29]. We evaluated the effect of the ICBT program on 11 somatic symptoms (e.g., “I have had lower back pain”; “I have lost my appetite”). This study was targeted at workers and BJSO was used for the purpose of eliciting their physical symptoms.

#### Statistical analysis

First, we conducted paired t-tests to compare the pre- and post-intervention POMS scores. Second, we calculated effect size (Cohen’s *d*) to assess the degree of change in significantly reduced POMS factor scores. Third, we conducted paired *t*-tests to compare the pre- and post-intervention somatic scores of the BJSQ. Fourth, we supplementarily conducted ANOVA to compare the pre- and post POMS-TA scores of participants who did and did not complete the tasks.

## Results

First, we examined the effect of the ICBT program on mood. We used paired *t*-tests to compare the pre- and post-intervention POMS scores and found a significant improvement in the POMS-TA subscale (*t*[47] = 2.06, *p* < .05). However, no significant difference was found for the POMS-D and POMS-F subscales (*t*[47] = −1.08, *p* = .28; *t*[47] = 0.19, *p* = .85, respectively; Table 2).

**Table 2:** Profile of Mood State (POMS) scores before and after the ICBT program

	Pre		Post		*t*-value (*df* = 47)
	Mean	SD	Mean	SD	
POMS TA (T scores)	49.31	8.15	47.44	8.54	2.06*
POMS D (T scores)	45.96	9.81	47.10	6.47	−1.08
POMS F (T scores)	45.88	9.11	45.71	7.72	0.19

*TA* tension-anxiety subscale, *D* depression subscale *F* fatigue subscale

*p < .05

We calculated effect size (Cohen’s *d*) to assess the degree of change in the POMS-TA scores. Cohen [30] categorized effect sizes as small (0.20–0.49), medium (0.50–0.79), and large (0.80 or more). The effect size for the POMS-TA subscale was *d* = 0.23 (95% confidence interval (CI) = −0.17 to 0.63); thus, according to Cohen’s classifications, the change in POMS-TA scores for the ICBT group was small.

To investigate somatic symptoms, the somatic scores on the BJSQ were assessed using paired *t*-tests. Low back pain was the only somatic symptom that improved significantly over the course of the program (baseline, 1.83 ± 0.95 vs. post-intervention, 1.56 ± 0.87; *p* = .03; Table 3).

**Table 3:** Comparison of pre-and post-intervention scores for somatic items on the Brief Job Stress Questionnaire

	Pre		Post		*t*-value (*df* = 47)
	Mean	SD	Mean	SD	
I have felt dizzy	1.33	0.63	1.25	0.53	0.85
I have experienced joint pains	1.23	0.52	1.31	0.62	−0.85
I have experienced headaches	1.58	0.65	1.50	0.65	0.81
I have had a stiff neck and/or shoulders	2.40	1.01	2.31	1.06	0.78
I have had lower back pain	1.83	0.95	1.56	0.87	2.22*
I have had eye strain	2.58	0.96	2.48	0.90	0.90
I have experienced heart palpitations or shortness of breath	1.21	0.46	1.25	0.60	−0.44
I have experienced stomach and/or intestine problems	1.75	0.96	1.75	0.84	0.00
I have lost my appetite	1.21	0.41	1.31	0.59	−1.40
I have experienced diarrhea and/or constipation	1.63	0.96	1.83	0.88	−1.49
I haven’t been able to sleep well	1.48	0.58	1.48	0.68	0.00

*p < .05

**Fig. 2:**
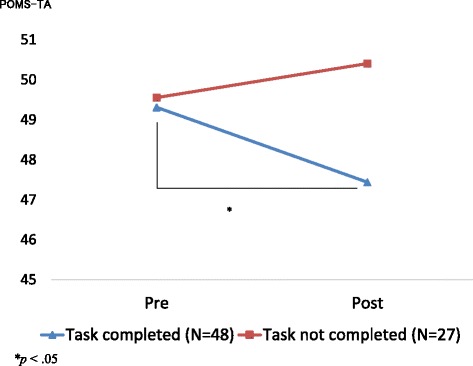
Comparison of the Profile of Mood States-tension-anxiety (POMS-TA) scores of participants who did and did not complete the tasks. We found a significant tendency toward an interaction between group and time of assessment for the POMS TA subscale (*F*(1, 73) = 2.94, *p* = .091). The Bonferroni post hoc test revealed that the POMS-TA scores improved significantly for the participants who completed the tasks (*p* < .05)

We conducted a supplemental analysis to compare the POMS-TA scores of participants who did and did not complete the tasks. ANOVA results (Fig. 2) revealed a tendency toward an interaction (*F*(1, 73) = 2.94, *p* = .091): the POMS-TA score was significantly improved for the participants who completed the task (*p* < .05).

## Discussion

We examined the effect of a brief ICBT program administered with a soft drink containing a supplement (L-carnosine) on healthy Japanese workers. The goal of the therapy was to relieve tension and anxiety. Additionally, a supplementary analysis suggested that completing the program tasks significantly improved tension and anxiety, highlighting the importance of compliance.

The effect size of the change in the POMS-TA score was small (*d* = .23), which is consistent with previous research showing that non-facilitated computerized CBT, which includes both Internet based and off-line software based programs, has a small effect (*d* = .38) [3]. Our ICBT intervention consisted of video clips, an e-textbook, and a supporting comic strip, available on demand. Although the duration of the intervention was short (4 weeks), the components were accessible and readily understood, and the program reduced tension and anxiety. Furthermore, the supplement drink may have improved the mental state of participants and increased their ability to complete the tasks. The supplement drink has been shown to improve health; moreover, continual consumption of the drink was an indication of the participants’ commitment to the ICBT program.

However, the completion rate of the ICBT program was relatively low. The autonomous self-help nature of the intervention may account for the low completion rate. An e-mail notification was automatically sent to all participants to remind them to complete each task; however, we provided no therapist contact or support staff, which may have decreased the motivation to complete all of the tasks.

Our finding that the intervention did not significantly affect fatigue and depression was not consistent with previous studies suggesting that foods and drinks containing L-carnosine promote recovery from mental fatigue [23, 24]. In a study conducted by Shimizu et al. [23], subjects consumed a supplement drink containing an anti-fatigue agent, imidazole dipeptides, for 8 weeks. The duration of our program was 4 weeks, which may have been too short for the supplement drink to affect fatigue or depression.

With regard to somatic symptoms, participants reported that our program significantly improved low back pain. Previous investigations have found differences in improvement of the anxiety of hypochondriacal patients with and without low back pain [31, 32]. Nakao et al. [31] reported that high anxiety at entry into the CBT program predicted a better treatment outcome for somatic symptoms and health anxiety associated with cognitive-behavioral interventions. The ICBT program improved the anxiety and low back pain of workers. Therefore, it may be that decreased levels of anxiety are related to improved low back pain.

Abbott et al. [33] investigated the effectiveness of an ICBT program for tinnitus distress and failed to find a significant difference in outcome between ICBT and information-only groups. However, the authors noted that the high attrition rate and small sample size, particularly in the intervention group, limited the generalizability of their findings. This highlights the importance of determining why participants drop out of internet-based programs to identify ways to increase motivation and compliance. In fact, we found that the change in the POMS-TA subscale was significant only in the group that completed the tasks, suggesting that high motivation and compliance are the keys to an effective brief ICBT program with a supplement drink.

## Limitations

Our study has several limitations. First, all of the subjects were tested under the same condition, i.e., they all participated in the ICBT program and consumed the supplement drink. Further study including subjects who take the supplement only and a control group will be necessary to clarify the effects of the supplement drink alone and together with ICBT. It is thought that these methods will also control the influence of “the Hawthorne effect”. Also, in this study, since we did not set a group to take a placebo drink, it is indicated as a subject to be studied in the future. Second, the present study took a completely self-help approach with no therapist or support staff contact. It is likely that outside guidance would increase engagement and compliance; thus, the potential effects of therapist support on our ICBT program should be examined. In terms of the dropout rates, the values of the present study were relatively high because of its complete self-help approach. Because our aim was a preliminary examination of the effect of the program tested in this research, we emphasized and analyzed only the data of participants who accomplished all of the tasks. For that purpose, we analyzed and examined only the data of the program completers. As for this point, future research will need to be done with a strict research design and that includes a control group or that uses intention to treat analysis to provide greater clarity. Third, our participants were relatively healthy employees. Future studies are needed to examine the effects of the brief ICBT program on individuals at high risk of mental health problems and those who have mental disorders. Additionally, the participants in the present study included those who belonged to the company that created the supplement drink. The participants may have implicitly reported desirable results, which may have biased the study.

## Conclusions

We investigated the effect on psychological well-being of a self-help ICBT program administered with a supplement drink containing L-carnosine. The combined program reduced the subjective experience of anxiety and low back pain in these workers. Future studies are needed to compare multiple groups and to examine the effect of therapist support.

## Abbreviations

ANOVA: Analysis of variance
CBT: Cognitive behavioral therapy
D: Depression
F: Fatigue
ICBT: Internet-based cognitive behavioral therapy
POMS: Profile of mood states
SD: Standard deviation
TA: Tension-anxiety
